# Estimating the tolerance of brachial plexus to hypofractionated stereotactic body radiotherapy: a modelling-based approach from clinical experience

**DOI:** 10.1186/s13014-021-01822-5

**Published:** 2021-06-07

**Authors:** Irina Kapitanova, Sharmi Biswas, Sabrina Divekar, Eric J. Kemmerer, Robert A. Rostock, Kenneth M. Forster, Rachel J. Grimm, Carla J. Scofield, Jimm Grimm, Bahman Emami, Anand Mahadevan

**Affiliations:** 1 Department of Psychiatry, Mount Sinai St. Luke’s Hospital, New York, NY USA; 2 Department of Pediatric Nephrology, Weill Cornell Medicine, New York, NY USA; 3 Sackler School of Medicine, Tel Aviv University, New York, NY USA; 4 Department of Radiation Oncology, Geisinger Cancer Institute, 100 N Academy Ave, Danville, PA 17822 USA; 5 Department of Radiation Oncology, Thomas Jefferson Hospital, Philadelphia, PA USA; 6 Department of Medical Imaging and Radiation Sciences, Thomas Jefferson University, Philadelphia, PA USA; 7 Department of Radiation Oncology, Loyola University Medical Center, Chicago, IL USA

## Abstract

**Background:**

Brachial plexopathy is a potentially serious complication from stereotactic body radiation therapy (SBRT) that has not been widely studied. Therefore, we compared datasets from two different institutions and generated a brachial plexus dose–response model, to quantify what dose constraints would be needed to minimize the effect on normal tissue while still enabling potent therapy for the tumor.

**Methods:**

Two published SBRT datasets were pooled and modeled from patients at Indiana University and the Richard L. Roudebush Veterans Administration Medical Center from 1998 to 2007, as well as the Karolinska Institute from 2008 to 2013. All patients in both studies were treated with SBRT for apically located lung tumors localized superior to the aortic arch. Toxicities were graded according to Common Terminology Criteria for Adverse Events, and a probit dose response model was created with maximum likelihood parameter fitting.

**Results:**

This analysis includes a total of 89 brachial plexus maximum point dose (Dmax) values from both institutions. Among the 14 patients who developed brachial plexopathy, the most common complications were grade 2, comprising 7 patients. The median follow-up was 30 months (range 6.1–72.2) in the Karolinska dataset, and the Indiana dataset had a median of 13 months (range 1–71). Both studies had a median range of 3 fractions, but in the Indiana dataset, 9 patients were treated in 4 fractions, and the paper did not differentiate between the two, so our analysis is considered to be in 3–4 fractions, one of the main limitations. The probit model showed that the risk of brachial plexopathy with Dmax of 26 Gy in 3–4 fractions is 10%, and 50% with Dmax of 70 Gy in 3–4 fractions.

**Conclusions:**

This analysis is only a preliminary result because more details are needed as well as additional comprehensive datasets from a much broader cross-section of clinical practices. When more institutions join the QUANTEC and HyTEC methodology of reporting sufficient details to enable data pooling, our field will finally reach an improved understanding of human dose tolerance.

## Background

Stereotactic body radiation therapy (SBRT) is a treatment option increasingly used for patients with lung cancer, including apical lung tumors, who are not surgical candidates. The main objective of the treatment is to provide the most effective SBRT dose on the tumor with minimal effect on normal tissue while avoiding post-radiation complications. Based on the tumor location (proximal of the brachial plexus), tumor size, dose, and numerous other factors, a potentially severe adverse effect after SBRT is radiation induced brachial plexopathy (RIBP) [1, 2]. Onset of RIBP symptoms may occur from months to years after the radiotherapy [3]. Brachial plexopathy, as defined in the Common Terminology Criteria for Adverse Events (CTCAE) v.5 [4], may include muscle weaknesses of the upper limbs, neuropathic pain, limitation of movement, paresthesia, and wasting. Understanding the tradeoffs between the benefits and risks in SBRT dose and fractionation can provide clarity by considering the range of severity in symptoms, from asymptomatic to full loss of movement of the upper extremity.

In 1991, the Emami paper [5] recommended a 5% risk in 5-year tolerance dose (TD 5/5) on the entire brachial plexus to be 60 Gy in conventional fractionation, based on expert opinion and on dose–response models [6]. Just 3 years later, the first clinical SBRT paper [7] included a dose–response model [8] to guide clinical practice, and a recent dose–response model for brachial plexus has been published [2] by the same institution. After a quarter of a century of SBRT practice, other studies validating these models are lacking and are needed to definitively determine tolerance of brachial plexus to SBRT. North American clinical trials for stereotactic ablative body radiotherapy (SABR) began at Indiana University [9], and the brachial plexus dose and toxicity outcome for each patient in a cohort was published [1]. The datasets from Indiana University and Karolinska Institute were pooled in the current study and analyzed as recommended by QUANTEC methodology [10, 11]. If this was standard practice in radiation oncology, then our understanding of human dose tolerance of various normal tissues to radiation would be vastly improved. Unfortunately, these examples are the extreme rarity, to the degree that, although a PubMed search of (SBRT OR SABR) AND (spinal cord) returns more than 250 papers, the High Dose per Fraction, Hypofractionated Treatment Effects in the Clinic (HyTEC) [12] effort was only able to find 3 papers that provided full datasets with critical structure dose and toxicity outcome per patient for spinal cord, which only represents about 1% of the published literature.

If detailed reporting of the spinal cord is so rare, even though it is among the most important critical structures in the body, it will be even harder to accumulate sufficient data for other organs. Therefore, is it possible to create comprehensive Emami-style dose tolerance limits [5] for intricate structures such as brachial plexus? The goal of the dose volume histogram (DVH) Risk Map [13] is to provide a modernized graphical view of Emami-style unified low- and high-risk limits, along with a numerical summary of the constraints and estimates of associated risk. The aim of this paper is to summarize initial steps towards creation of the DVH Risk Map for the brachial plexus as an impetus to improve data reporting across published literature for better understanding of tolerance levels.

## Methods

To identify brachial plexus dose tolerance after SBRT based on dose–response models of clinical outcomes data, the following 6 elements are needed: (1) dose to the brachial plexus, (2) fractionation, (3) volume, (4) endpoint, (5) follow-up time, and (6) incidence of the endpoint occurring within the follow-up time [13]. These 6 items are needed per patient, or at least in enough detail to stratify data into small groups of patients with similar characteristics. A PubMed search for (brachial plexus) AND (stereotactic OR SABR OR SBRT) was performed, and 52 papers were found as of July 2020, but only two of the studies came close to providing the needed information for all patients in a study.

The two datasets were comprised of patients treated (1) at Indiana University and the Richard Roudebush Veterans Administration Medical Center from 1998 to 2007 [1] as well as (2) the Karolinska Institute from 2008 to 2013 [2]. All patients in both studies were treated for apically located lung tumors localized superior to the aortic arch. A total of 89 patients (with 93 lesions) from both institutes received SBRT and were included in this analysis.

Physical dose without any biological conversions was used in the graph of presented brachial plexus maximum doses in the Indiana dataset, and the linear quadratic (LQ) model [14, 15] as well as the universal survival curve (USC) [16] were used to assess the data. In the Karolinska dataset, dose–response models were created using both the LQ and USC models. The probit dose–response model [17] was used in the Lindberg et al. [2] study, so this model was also used in our pooled analysis for consistency. Brachial plexus maximum point dose (Dmax) values were digitized from the source graphs [1, 2] with the DVH Evaluator software [13], which then was used to perform maximum likelihood parameter fitting [18] to determine the values for the probit model [17], and confidence intervals were constructed using the profile likelihood method [19, 20].

All clinical data were collected from the patient records and graded using the Common Terminology Criteria for Adverse Events (CTCAE). Only toxicities of Grade 2 and greater in both studies were scored as complications. Indiana University used CTCAE version 3.0 [21] with scoring of grade 1–4 while Karolinska used CTCAE version 4.0 [22]. CTCAE version 3.0 focused more on the symptoms affecting activities of daily living while version 4 stressed the severity of the symptoms. For the purpose of inclusion, we have also included the Modified Late Effects Normal Tissue—Subjective Objective Management Analytic (LENT-SOMA) scale [23, 24] to compare the brachial plexus adverse effects. The details of the grading of toxicity are shown in Table 1. The following variables were considered in the comparison of toxicity rates: gender, age, histology, number and size of tumors, dose of SBRT, number of fractions, and time to brachial plexopathy from SBRT. The Fisher Exact Test was used to assess significance among individuals with toxicity and those without toxicity [25, 26].

**Table 1:** Endpoint definitions: brachial plexus toxicity grading scales

	CTCAE Version 3.0 [21]	CTCAE Version 4.0 [22]	Modified LENT-SOMA scale [23]
Grade 1	Asymptomatic brachial plexopathy	Asymptomatic effects	Mild sensory deficits, no pain, no treatment required
Grade 2	Symptomatic brachial plexopathy without interfering with activities of daily living (ADL)	Moderate symptoms limiting ADL	Moderate sensory deficits, tolerable pain, mild arm weakness
Grade 3	Symptomatic brachial plexopathy and interfering with ADL	Severe symptoms limiting self care ADL	Continuous paresthesia, with incomplete paresis, pain medication required
Grade 4	Disabling brachial plexopathy	N/A	Complete paresis, excruciating pain, muscle atrophy, regular pain medication required

*CTCAE* common terminology criteria for adverse events, *LENT* late effects normal tissues, *SOMA* subjective, objective, management, analytic, *ADL* activities of daily living

## Results

Patient characteristics, SBRT doses, and grading of radiation induced brachial plexopathy are compared in Table 2 for both studies. The median patient age was 72 and 73 for Karolinska University and Indiana University, respectively. 93 tumors were treated in total with 22 patients having metastases.

**Table 2:** Apical lesion patient characteristics

	Indiana University	Karolinska University	Total
Number of patients	37	52	89
Gender			
Male	21	23	44
Female	16	29	45
Age at treatment, median (range)	73 (57–81)	72 (35–88)	
Number of tumors	37	56	93
Primary lung cancer (NSCLC)	37	30^a^	67
Metastases	0	22	22
Tumors			
Right	21	28	49
Left	16	28	44
Volume cc, median (range)	GTV 13 (1–113)	CTV 9.1 (0.10–74.5)	
Follow-up months, median (range)	13 (1–71)	30 (6.1–72.2)	
Total treatment dose (Gy), median (range)	57 (30–72)	Median 45 Gy in 3 fx BED10 Range: 95–138 Gy	
Median dose per fraction (Gy) (range)	19 (10–24)	15 (6–17)	
Median number of fractions (range)	3 (3–4)	3 (3–10)	
Number of patients with brachial plexopathy			
Total	7	7	14
Grade 2	4	3	7
Grade 3	2	4	6
Grade 4	1	0	1
Brachial plexopathy development in months post-SBRT, median (range)	7 (6–23)	5.8 (0.7–13)	

*NSCLC* non-small cell lung cancer, *GTV* gross tumor volume, *CTV* clinical tumor volume, *fx* fractions; *BED10* biological effective dose with α/β = 10 Gy

^a^One patient with metastasis later on

### Dose, fractionation, and volume of the brachial plexus

At Indiana University, the median prescribed treatment dose was 57 Gy in 3–4 fractions and the maximum brachial plexus dose ranged from 6 to 83 Gy (median, 26 Gy). The Indiana University dataset had 37 brachial plexus Dmax values (for 36 patients) that were all included in the model. The paper did not report which patients received 3 or 4 fractions, or volume information, and these are the main limitations of the study [1]. Both published datasets [1, 2] used biological conversions with α/β = 3 Gy, thus the biological effective dose is denoted as BED_3_. According to the linear quadratic model [14, 15], the 2 Gy per day equivalent EQD2 = 60 Gy Emami brachial plexus limit [5] corresponds to BED_3_ = 100 Gy. In 3 fractions, LQ equates this to 26 Gy, which was equal to the median brachial plexus Dmax of the 37 cases, and this was initially used as a cutoff point of risk analysis, finding the two-year Kaplan–Meier risk of 46% vs 8% above and below this cutoff [1].

The Karolinska group used 45 Gy in 3 fractions for 80% of the cases, therefore that also was the median prescription. One patient was treated with 60 Gy in 10 fractions, six were treated with 56 Gy in 8 fractions, and the rest were in 3–5 fractions. The authors performed analysis with both USC and LQ models and found no major difference between the two for their data, so presented the data in terms of BED_3_ with the LQ model. Brachial plexus Dmax ranged from BED_3_ = 0.10–524 Gy, which we converted to 3-fraction equivalent dose since the median number of fractions in both studies was 3. The Karolinska dataset presented model parameters for Dmax, in addition to dose to hottest X cc (Dx) for D0.1cc, D1cc and D3cc, but the group from Indiana University only reported on Dmax. Therefore, the pooled model has no volume information, and consists of maximum point doses only.

### Endpoint, Follow-up time, and estimated risk of the endpoint occurring within the follow-up time

Follow-up was longer in Karolinska with median 30 months (range 6.1–72.2) while Indiana had a median of 13 months (range 1–71). Among the 89 patients included in both studies, 14 of them developed CTCAE grade 2 or higher RIBP, acknowledging the differences among the endpoint definitions in Table 1. Among the 14, the most common complications were grade 2, comprising 7 patients. Only 1 patient from Indiana University was recorded with grade 4 disabling RIBP described as shoulder ache progressing to paresthesia and further worsening to arm and hand wasting. This case corresponded to brachial plexus Dmax of 76 Gy. One patient from Karolinska also noted signs of RIBP 13 months post SBRT further progressing to total paralysis of the arm, but was scored as grade 3 since CTCAE 4.0 is without grade 4 RIBP. Therefore, the LENT-SOMA scale is a useful point of comparison in this regard as shown in Table 1, because it does include a definition of grade 4.

**Fig. 1:**
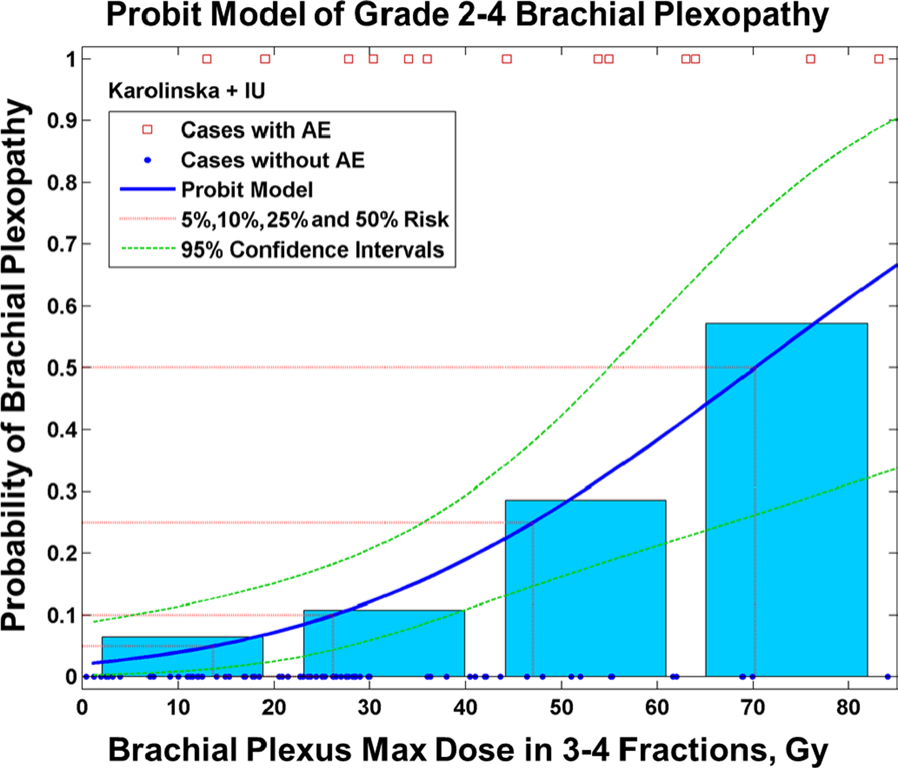
The probit model [17] of Grade 2–4 Brachial Plexopathy shows the Dmax values of the Karolinska and Indiana University (IU) datasets [1, 2] with red squares denoting the cases corresponding to CTCAE grade 2 or higher adverse events (AE), blue dots representing the cases without AE, and quartiles plotted as the four blue bars. According to the model in 3–4 fractions, the risk of a brachial plexopathy with the dose of 26 Gy is 10%, whereas the 25% and 50% risk levels correspond to 47 and 70 Gy respectively

It is also important to note that in the Karolinska study, 13 patients underwent additional radiotherapy to the lung ipsilateral to the tumor site that is not included in the model in Fig. 1. Out of the 13, 10 of the patients had very low additional brachial plexus dose, D_max_ BED_3_ ≤ 3.1 Gy. The remaining 3 had a prior conventional dose of D_max_ BED_3_ = 90–123 Gy with only 1 patient from this subset developing RIBP. Therefore, for the Karolinska study, 6 out of 7 patients developed RIBP strictly only from the SBRT.

### Dose–response model and DVH Risk Map

Given the approximation of the 6 elements needed for a dose–response model [13], and considering their limitations, caveats, and confounding factors as enumerated above and described in the discussion, a pooled dose–response model was created. According to the fitted probit model [17–20], the dose corresponding to 50% risk of complications was 70.2 Gy (95% CI 55–116 Gy), and the slope parameter at this dose was 0.49 (95% CI 0.35–0.74). The probit model and 95% confidence intervals are depicted in Fig. 1 [17–20]. Significance was assessed via the Fisher Exact Test [25, 26] split at the median dose of the Indiana dataset (Dmax = 26 Gy), and at the median dose of the combined dataset (Dmax = 27 Gy), yielding p-values of 0.01 and 0.0035, respectively. The 5%, 10%, and 25% risk levels were 13.7, 26, and 47 Gy, respectively, in 3–4 fractions. Appendix Fig. 5 shows that for this dataset, probit and logistic models are within ± 1.6% of their average, up to 60 Gy in 3–4 fractions, and diverge from each other above this dose where the data is very sparse.

**Fig. 2:**
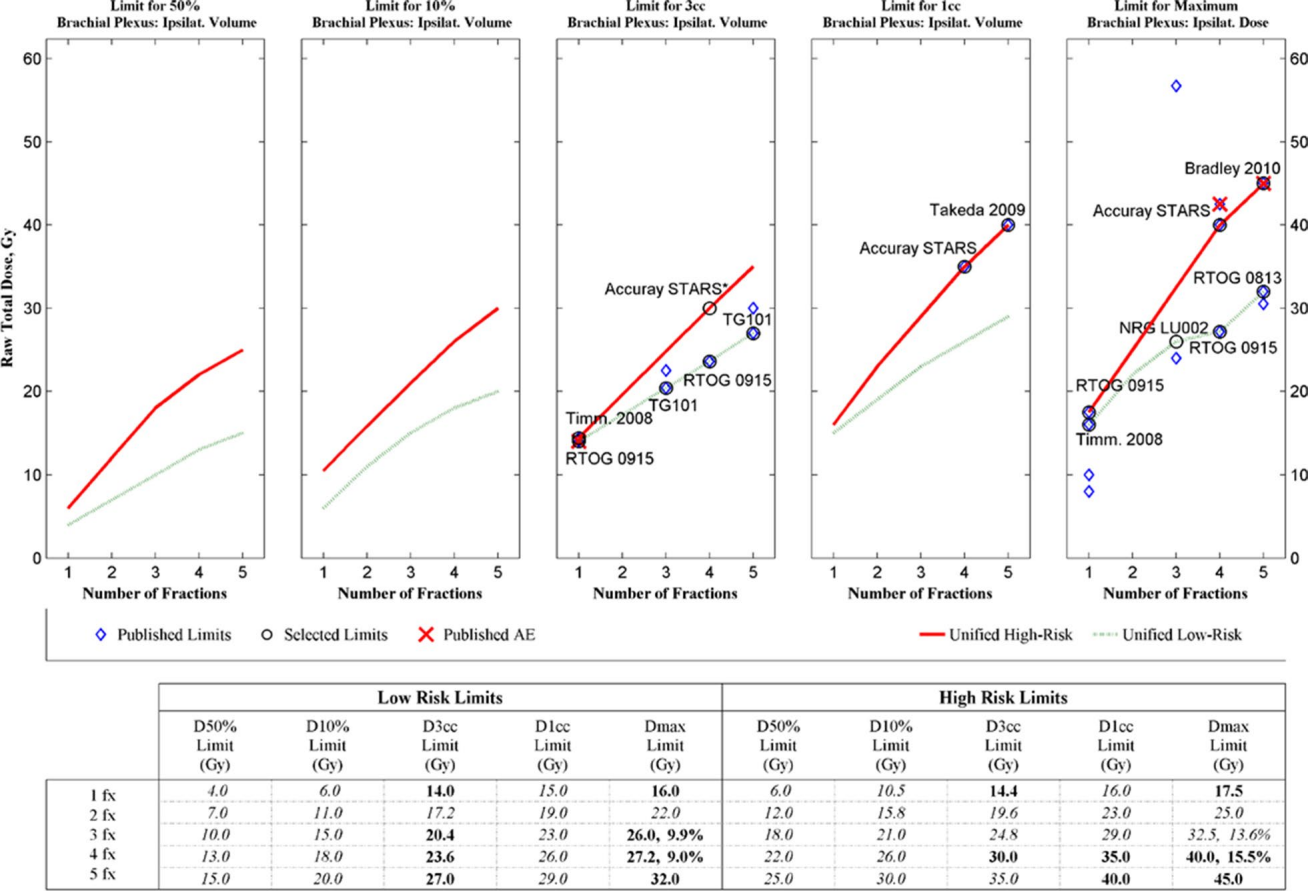
DVH Risk Map for brachial plexus. Note that NRG LU-002 protocol has adopted the Dmax = 26 Gy in 3 fraction constraint from Forquer 2009 [1], which we have designated as the low-risk limit for Dmax in 3 fractions. In the tabular portion of the figure, the limits that had already been published are bold, and the rest are italicized. For the Dmax limits in 3–4 fractions, the estimated risk level interpolated from the model in Fig. 1 is shown to the right of the dose constraint

The connection between dose/volume, fractionation, and incidence of complications for the endpoint of grade 2 or higher brachial plexopathy is summarized in the form of a DVH Risk Map [13] in Fig. 2. This map includes a graph of published dose constraints in the upper portion of the figure, as well as a numerical summary of low- and high-risk constraints in the lower portion of the figure, with the resultant estimates of risk from the pooled model from Fig. 1. Appendix Fig. 4 shows how the 5% and 50% risk levels at 5 years (TD 5/5 and TD 50/5) in the Emami paper [5] were obtained from expert opinion and models in the Burman paper [6]. Similarly, risk levels in the DVH Risk Map in Fig. 2 are interpolated from the dose–response model of Fig. 1. A more complete description of the DVH Risk Map may be found for several other organs-at-risk in the literature [27–29].

The DVH Risk Map in Fig. 2 shows the number of fractions on the x-axis and the raw total physical dose without any BED conversion on the y-axis. Each of the five panels specifies a dose/volume metric including dose for the 50% and 10% volumes, as well as D3cc, D1cc, and Dmax. Published dose constraints from Appendix Table 3 are plotted as blue diamond marks on the map (Fig. 2). These constraints were partitioned into low- and high-risk categories from among the more established limits, represented as the circled selected limits with labels. The red X represents the dose at which a published Adverse Event (AE) occurred, as may be seen in Appendix Table 3. For visualization, a trendline of low- and high-risk are drawn as the dashed green and solid red lines in this map. Although the partitioning is somewhat arbitrary, this is approximately analogous to the TD5/5 and TD 50/5 Emami limits for conventional fractionation, but now customized to the published limits in a more useful clinical range of practice. Based on the pooled dataset, as may be seen from the tabular portion of Fig. 2, the low-risk trend of brachial plexus Dmax in 3–4 fractions is about 10% risk and the high-risk trend is about 15% risk.

## Discussion

**Fig. 3:**
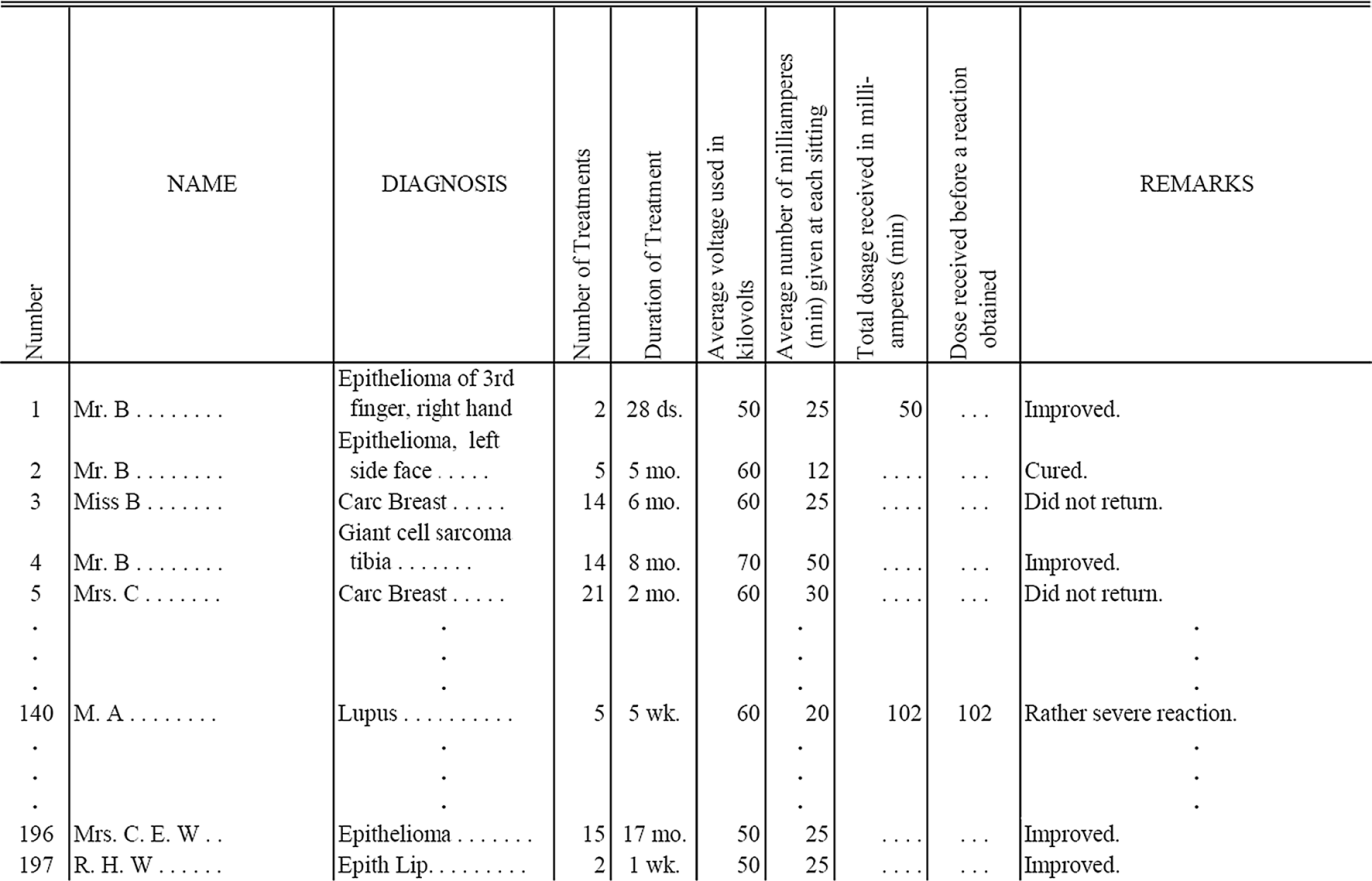
Excerpt of an example de-identified published database from 1914, “Some Experiments in Standardization of Dosage” [33]. Although precision is limited since this table pre-dates the definition of the rad by 4 decades [35] and was long before any of the modern grading systems [4], nevertheless the sharing of fractionation, multiple parameters of dose, and outcome per patient as still recommended by HyTEC and QUANTEC [10–12, 32] is truly remarkable, since this dataset of 197 cases is from more than 100 years ago

Bias and uncertainty can result from single institution non-randomized heterogeneous mixtures of patients with varying follow-up times and unknown censoring of competing risks. Throughout the past quarter of a century, over a million patients have been treated with radiosurgery on Gamma Knife alone [30], over a million more patients have been treated with SBRT on CyberKnife alone [31], and countless more have been treated on stereotactically capable linear accelerators. No excuse remains for there to be only two limited published datasets for an important critical structure like the brachial plexus. It is imperative that the field of radiation oncology collects data more rigorously as highlighted by the lessons of QUANTEC [10, 11] and as continues to be emphasized by all the HyTEC papers [12, 32]. In the meantime, it is important to glean as much information as possible from the sparse datasets that do exist, and to pool them into increasingly larger datasets [10]. A full de-identified database of 197 patients with dosimetric information and outcome for each patient was published more than 100 years ago [33], showing that it is possible to accomplish this without sophisticated algorithms (Fig. 3). One of the first dose–response models was created more than 90 years ago from clinical data by hand on graph paper [34], even before the first electronic computer was invented. With modern automated algorithms, there is no excuse to not save and analyze the data in properly designed studies with actuarial outcomes at specific time points in multiple institutions with large cohorts of data. The dose-tolerance numbers for conventional fractionation from the Emami paper were based on expert opinion over 30 years ago, in terms of the radiation dose limits for 1/3, 2/3 and 3/3 organ volume, with the probability of 5% (TD 5/5) or 50% (TD 50/5) risks of complications within a 5-year follow-up. The original paper did emphasize the need for more research and available data. Two decades later the ensuing accumulated published data was consolidated into QUANTEC [36] which was much more accurate owing to the growing body of cooperative trials and institutional studies. However, the improved accuracy of QUANTEC also came with increased complexity and varied format of the limits, which is difficult to use in day to day clinical work. The goal of the DVH Risk Map [13] is to balance the convenience of a unified framework of dose tolerance limits in low-risk and high-risk categories, with the accuracy of dose–response modeling from all the emerging published clinical data, particularly in the setting of hypofractionated SBRT.

Brachial plexus dose tolerance for conventional fractionation has been studied [5, 37, 38] and contouring guidelines are available [2, 39, 40]. The Emami limit for brachial plexus of EQD2 = 60 Gy [5] corresponds to 26 Gy in 3 fractions, which is remarkably the same dose limit as recommended in the Indiana study [1]. However, the paradigm has transformed from allowing 100% organ exposure at that dose in conventional fractionation [5], now all the way down to the 0% volume at the same dose for SBRT [1, 41].

About one third of the combined dataset had Dmax values in excess of 10 Gy per fraction, where the LQ model has been questioned [16]. For this reason the Karolinska authors compared LQ to USC, and found no major difference for this data [2]. The Indiana dataset was published in terms of physical dose, which avoids questions regarding BED models, but is itself a major limitation of the pooled model since the fractionation was not reported per patient.

Gender, age, histology, number and size of tumors, dose of SBRT, number of fractions, and time to brachial plexopathy from SBRT varied but were reasonably similar across studies as may be seen in Table 2. However, neither study provided these values per patient, therefore no multivariate analyses or subgroups of dose–response models could be performed. The median length of patient follow-up was more than twice as long in the Karolinska study (30 vs 13 months), but at least the median follow-up in the Indiana University study was longer than the median onset of brachial plexopathy in either study (7 and 5.8 months). Both studies included some patients with less follow-up time than the latest reported complication in either study, so it is highly likely that a longer follow-up period would reveal at least somewhat higher percentage of complications in either study.

Limitations of both studies include data based on a small cohort of patients with limited follow-up. These data may not reflect the full incidence of toxicity after SBRT because many patients might not survive long enough for toxicity to develop or may be lost to follow-up for a variety of reasons. Another limitation is the usage of re-irradiation for some of the Karolinska cases, although this only caused one of the complications, so insufficient data were available to construct a model that could account for re-irradiation tolerance. The Karolinska authors reported distance and overlap of the brachial plexus to the tumor, but the Indiana University authors did not, so this factor was not included in the pooled analysis. Differences in grading of complications was acknowledged, which may contribute to inaccurate causal analysis. Half of the complications were grade 2, and only one potentially grade 4 paresis was reported in each of the two studies. However, the studies did not indicate the specific grade for each Dmax value of the whole dataset, so separate models for each grade cannot be created, as was done in a brain dose tolerance study [42]. Furthermore, as noted in Table 1, the grading scales vary especially for the higher-grade events. A risk of 10% is higher than ideal for brachial plexus, but until the grade of each patient is reported in a consistent scale, clinicians must use their own judgement when interpreting the results.

## Conclusions

For lung cancers near the apical region, brachial plexopathy is a major concern for high-dose radiation therapy. Based on our analysis of published data, the risk of grade 2 or higher brachial plexus toxicity after SBRT is approximately 5%, 10%, and 50% at 13.7, 26, and 70 Gy, respectively, in 3–4 fractions, but the risk of grade 3 or 4 toxicity remains unknown. This paper is not intended to be a final answer, but rather an appreciation of recent efforts and a plea for more data: it is commendable that the Indiana and Karolinska authors published the data that enabled this pooled model, as recommended by QUANTEC and HyTEC. When more institutions join the QUANTEC and HyTEC methodology of reporting sufficient details to enable data pooling, our field will finally reach an improved understanding of human dose tolerance.

## Figures and Tables

**Fig. 4:**
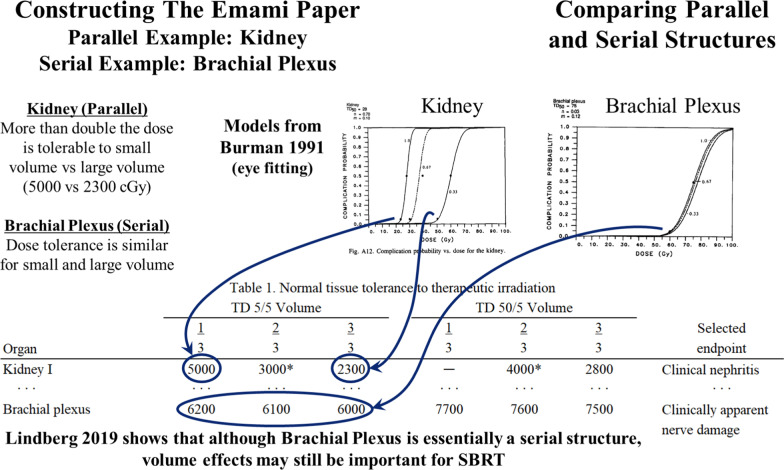
Emami paper [5] examples from expert opinion and Burman [6] models. Arrows depict how the 5% risk levels were interpolated from the Burman [6] model for the Emami [5] table of dose tolerance. Dots on the graph depict the TD 5/5 and TD 50/5 tolerance doses, which correspond to the values in the table. Kidney is a parallel structure so it is intuitive that the 5% risk level for 1/3 and 3/3 volumes were very different, whereas brachial plexus is predominantly a serial structure, so the 5% risk levels were fairly similar for conventional fractionation regardless of volume. However, Table 3 of the Karolinska study [2] shows a 50% reduction in tolerance of brachial plexus D3cc as compared to the maximum point dose (Dmax), therefore volume effects may be more important for SBRT. In the Emami paper [5], for both kidney and brachial plexus the TD5/5 and TD50/5 limits were in close agreement with the models. However, for other structures such as Bladder there was more reliance on expert opinion, as can be observed by the location of the TD 5/5 and TD 50/5 dots in the Burman paper [6], in relation to the modeled curves

**Fig. 5:**
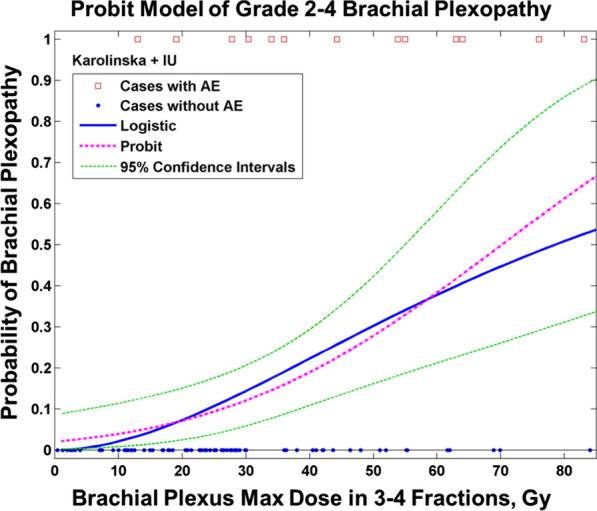
Comparison of logistic [74] and probit [17] in Schultheiss et al. [75] showed that for some datasets these two models may differ by less than 1% over their entire range. For this dataset as shown above, the two models differ by no more than ± 1.6% from their average, up to 60 Gy in 3–4 fractions, but diverge at doses above that where the data is sparse. *Abbreviations* *IU* Indiana University, *AE* adverse event (brachial plexopathy)

## Data Availability

The datasets generated during and/or analysed during the current study are available in the published literature.

## Appendix

See Figs. 4, 5 and Table 3.

**Table 3:** Published dose constraints that are plotted in the DVH Risk Map in Fig. 2 of the paper

# Fractions	Volume cc	Volume %	cc or % limit Gy	Max limit Gy	Mean limit Gy	Approx # AE ≥ G3	Approx # Pts dose	# Pts in study	Refs	Notes
1	5		14.4			0		33	[43, 44]	RTOG 0631, limit is for sacral plexus
1	0.03		18			0		33	[43, 44]	RTOG 0631, limit is for sacral plexus
1	3		14						[43, 44]	RTOG 0631
1	3		14			1		33	[44]	One G3 possible radiosurgery-related AE (neck pain)
1	0.03		17.5						[43, 44]	RTOG 0631
1	0.03		17.5			1		33	[44]	One G3 possible radiosurgery-related AE (neck pain)
1				17.5					[45, 46]	RTOG 0915, TG101
1				16					[47]	Also for sacral plexus
1	3		14.4						[47]	Also for sacral plexus
1	3		14						[45, 46]	RTOG 0915, TG101
1				10		0		23	[48, 49]	
1				8		0		32	[50]	
3				56.7					[51]	RTOG 1021 acceptable deviation
3				27					[52]	
3				26		7		37	[1, 53]	100 Gy BED3, 14% risk, 46% above, 8% below
3				24					[46, 47, 51, 54–65]	RTOG 0618, 1021, Accuray STARS, Also for sacral plexus, TG101
3				24		0		10	[56]	
3				24		0		38	[52, 62]	
3				24		0		79	[63]	
3				24		0		70	[65]	
3	3		22.5						[47]	Also for sacral plexus
3	3		20.4						[46, 51]	TG101, RTOG 1021
4				42.5		1	1	27	[66]	20% of brachial plexus > 40gy in one pt who had G3 AE
4		20%	40			1	1	27	[66]	20% of brachial plexus > 40gy in one pt who had G3 AE
4				40		1		27	[53, 59, 66, 67]	Accuray STARS, 1 AE at 40 Gy described in Chang 2015
4	1		35					27	[59, 66]	Accuray STARS
4	10		30					27	[59, 66]	Accuray STARS
4				26					[1, 53]	This limit has been attributed to 3–4 fractions
4				35		3	9	82	[68]	
4	0.2		30			3	7	82	[68]	
4				32					[52]	
4				27.2					[45, 69]	RTOG 0915
4	3		23.6						[45]	RTOG 0915
5				45		1		12	[63]	
5				35					[52]	
5	1		40					50	[70]	
5				32					[47, 71]	RTOG 0813, QOD, Also for sacral plexus
5				30.5					[46]	TG101
5				30					[72]	
5				29.6					[69]	
5	3		30						[47, 71]	RTOG 0813, QOD, Also for sacral plexus
5	3		27						[46]	TG101
7				35					[72]	
8				37		0		46	[73]	

*cc* cubic centimeters, *Approx* approximate, *AE* adverse event (brachial plexopathy), *G3* grade 3 or higher, *Pts* patients, *Rx* received, *Refs* references, *RTOG* radiation therapy oncology group, *TG* task group, *QOD* every other day, *BED* biological effective dose

The following form of the probit model [17] was used in the manuscript:

1 $$NTCPprobit = \frac{1}{{\sqrt {2\pi } }}\mathop \smallint \limits_{ - \infty }^{t} e^{{ - x^{2} /2}} dx$$

where *TD*_50_ is the 50% risk level, $t = \left( {D_{max} - TD_{50} } \right)/\left( {m \times TD_{50} } \right)$, and *m* is the normalized slope.

The following form of the logistic model [74] was used in Fig. 5 for comparison:

2 $$NTCPlogistic = 1/(1 + (TD_{50} /D_{max} )^{ \wedge } (4*g_{50} )),$$

where *TD*_50_ is the 50% risk level and *g*_50_ is the slope parameter.
